# Case report: The smallest 9p21.3 microdeletion involving *CDKN2A* but not *CDKN2B* causes multiple plexiform neurofibromas

**DOI:** 10.3389/fonc.2025.1437093

**Published:** 2025-02-19

**Authors:** Yuanyuan Zhang, Xiang Li, Haiming Gao, Chuang Qiu, Xiaoliang Liu

**Affiliations:** ^1^ Department of Clinical Genetics, Shengjing Hospital of China Medical University, Shenyang, China; ^2^ Department of Breast Surgery, Cancer Hospital of China Medical University, Cancer Hospital of Dalian University of Technology, Liaoning Cancer Hospital & Institute, Shenyang, China; ^3^ Department of Orthopedics, Shengjing Hospital of China Medical University, Shenyang, China; ^4^ Liaoning Key Laboratory of Reproductive Health, Liaoning Research Institute of Birth Health and Development, Reproductive Hospital of China Medical University, Shenyang, China; ^5^ Shenyang Key Laboratory of Biomedical Polymers, Liaoning Research Institute of Birth Health and Development, Reproductive Hospital of China Medical University, Shenyang, China

**Keywords:** 9p21.3 deletion, plexiform neurofibromas, genetic counseling, case report, CDKN2A

## Abstract

Chromosome 9p21.3 is a locus associated with a rare autosomal dominant cancer predisposition syndrome characterized by early-onset melanoma and a broad spectrum of neural system tumors. Two major tumor-suppressor genes, cyclin-dependent kinase inhibitor 2A and 2B (*CDKN2A* and *CDKN2B*), as well as a large non-coding RNA *ANRIL*, are often co-deleted in the core region. Herein, we report a pregnant woman who had developed more than 20 plexiform neurofibromas since the age of 13 and experienced 11 times of surgical resections. No melanoma or other tumors were found. A germline 9p21.3 deletion involving *CDKN2A* and the first exon of *ANRIL*, but not *CDKN2B*, was identified by whole exome sequencing (WES) and confirmed by quantitative PCR. Prenatal diagnosis was performed through copy number variation-sequencing (CNV-seq), and the pregnancy was terminated with informed choice for an affected fetus. All the eight cases carrying germline 9p21.3 deletions were reviewed for genotype–phenotype correlation, showing that our case with the smallest deletion had plexiform neurofibroma only, and the two cases of Eastern Asian origin had no melanoma. Our data highlight 9p21.3 deletion as a potential differential diagnosis for neurofibroma and emphasize the importance of CNV analysis on the WES data wherein small deletions might be easily overlooked.

## Introduction

Chromosome 9p21.3 is a locus associated with a rare autosomal dominant cancer predisposition syndrome characterized by early-onset cutaneous melanoma and a broad spectrum of neural system tumors. Two major genes *cyclin-dependent kinase inhibitor 2A* and *2B* (*CDKN2A* and *CDKN2B*) locate contiguously at 9p21.3, encoding p16^INK4A^ and p14^ARF^ by *CDKN2A* in an alternative reading frame, and p15^INK4B^ by *CDKN2B* (1). These encoding proteins are tumor suppressors that prevent cell-cycle progression synergistically through retinoblastoma (Rb) and p53 pathways (2). In addition, a large *ANRIL* (*CDKN2B-AS1*) with sequences overlapping *CDKN2B* and the upstream of *CDKN2A* is transcribed at the antisense direction into non-coding RNA to modulate the susceptibility to various tumors through transcriptional repression of *CDKN2A* and *CDKN2B* (3, 4). Other adjacent genes including the *methylthioadenosine phosphorylase* (*MTAP*) and a small antisense non-coding RNA *CDKN2A-DT* are frequently co-deleted with undetermined effects in tumorigenesis.

Up to now, only seven cases carrying germline 9p21.3 deletions have been reported, with variable deletion sizes and a tumor spectrum (3, 5–10). Here, we report a pregnant woman with germline 9p21.3 heterozygous deletion involving *CDKN2A*, *CDKN2A-DT*, and the first exon of *ANRIL*, who presented with only multiple plexiform neurofibromas (PNFs). This is thus far the smallest 9p21.3 deletion without affecting *CDKN2B*, and the first prenatal diagnosis and reproductive counseling for such affected individuals.

## Case report

**Figure 1:**
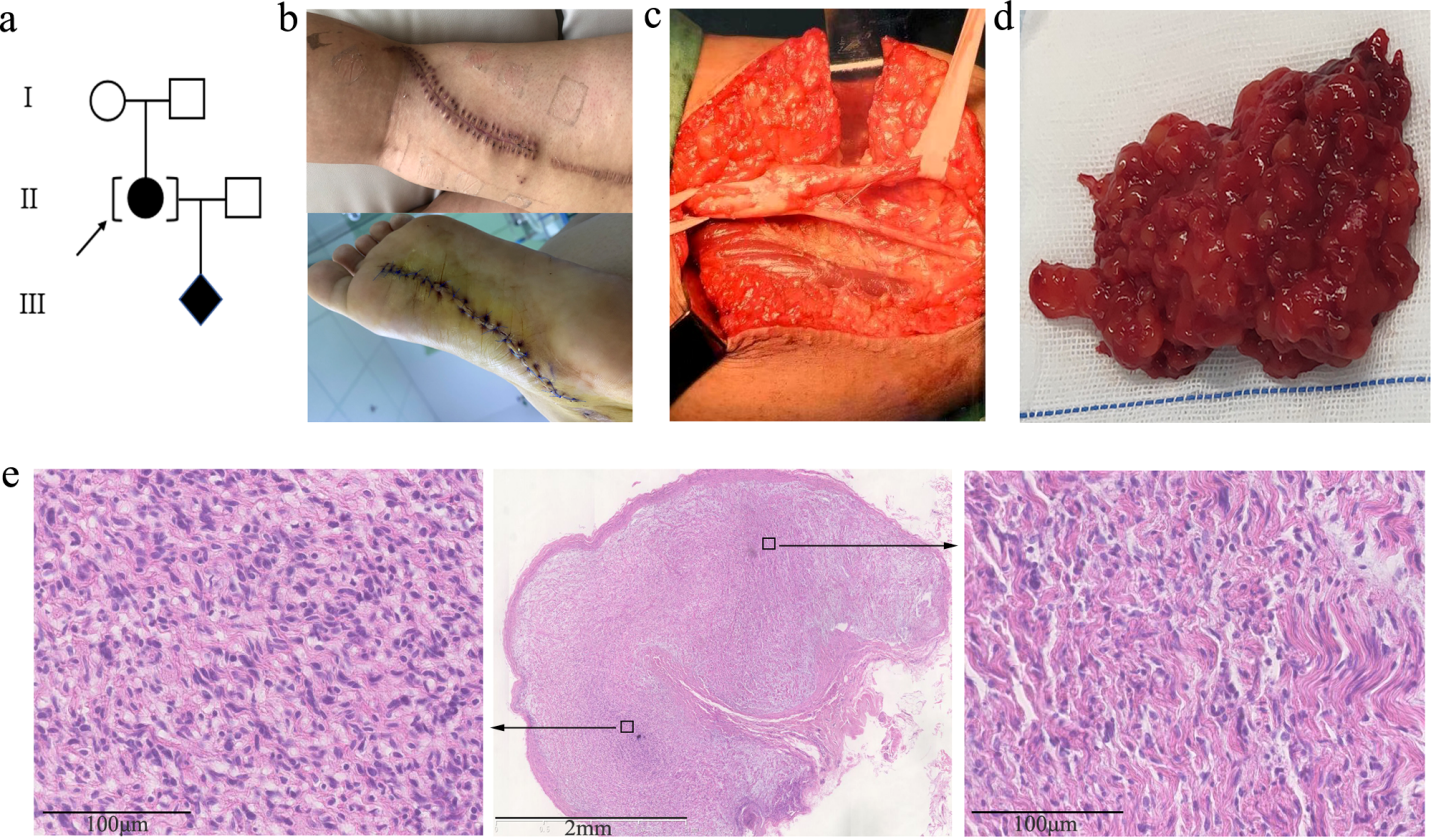
**(A)** Family pedigree of the case. **(B)** Photos of recent surgical incision sutures on the left leg and sole. **(C)** Tumor resection from the left sciatic nerve during surgery. **(D)** Representative gross plexiform neurofibromas tissue excised from the left leg of the proband. **(E)** Low (2.5×)-magnification image (middle) of histopathology by HE staining. The perineurium can be seen around the plexiform nodules. High (20×)-magnification images of local high (left) and low (right) cell density areas are framed with black squares and displayed by the black arrows. The tumor cells are spindle-shaped, with wavy nuclei and loose stroma, which is accompanied by obvious mucinous degeneration.

A 31-year-old gravida 1, para 0 Chinese woman (body weight: 62 kg, height: 162 cm) was referred to genetic counseling at 14 weeks of gestation because of multiple biopsy-proven neurofibromas. She was adopted with unknown familial history (**Figure 1A**). The first two neurofibromas were found on her left calf at the age of 13, with diameters of 2.5 cm–3.0 cm. Subsequently, the number of neurofibromas increased by 1 or 2 each year and was up to more than 20 by the time of referral. Most of the tumors were subcutaneous centered at the left lower limb, and only one tumor was deep in the pelvic cavity. She had undergone 11 times of surgical resections of these neurofibromas (representative photos shown in **Figures 1B-D**). The pathological examination supported typical features of PNF (**Figure 1E**). No other clinical indications of neurofibromatosis type 1 (NF1), including cafe-au-lait spots, axillary freckling, or inguinal freckling, were found by physical examination or cutaneous Woods lamp examination, and no Lisch nodule was identified by ophthalmological examination.

**Figure 2:**
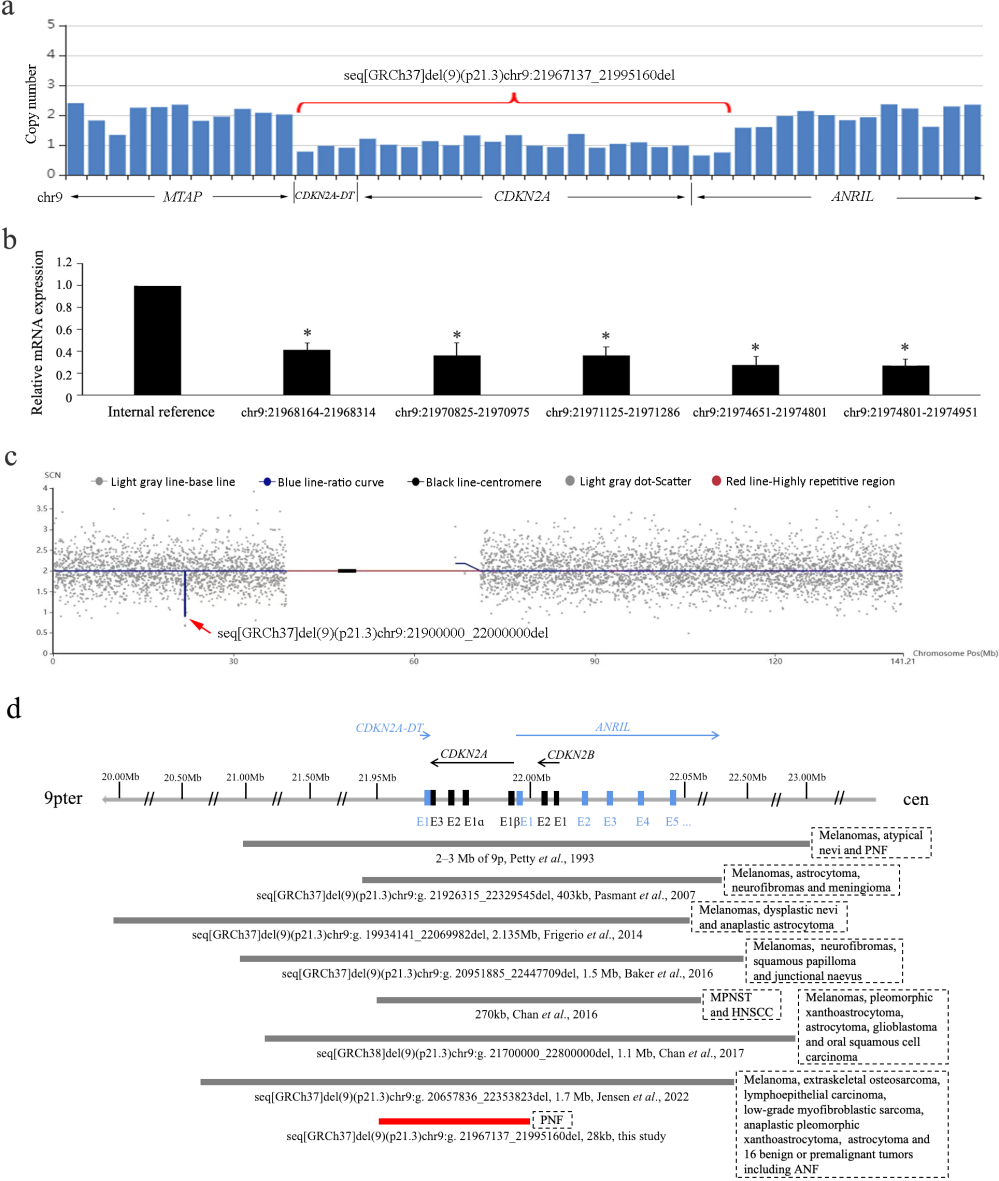
**(A)** Copy number loss of *CDKN2A, CDKN2A-DT*, and exon 1 of *ANRIL* by captured CNV analysis of the WES data. **(B)** Haploinsufficiency of *CDKN2A* gene by quantitative PCR using five pairs of primers. Data are collected in triplicate. *P < 0.05 in the ANOVA compared with internal reference. **(C)** Microdeletion at chromosome 9p21.3 spanning 21,900,000 to 22,000,000 by CNV-seq. The blue line indicates the ratio curve of the copy number in the patient, and the deletion is highlighted by a red arrow. **(D)** Schematic diagram of the reported germline 9p21.3 microdeletions involving the *CDKN2A/CDKN2B/ANRIL* locus. The bar lengths represent the deletion sizes. Our case is the smallest one not involving *CDKN2B* (indicated in red). PNF, plexiform neurofibromas; ANF, atypical neurofibromas; MPNST, malignant peripheral nerve sheath tumor; HNSCC, head and neck squamous cell carcinoma.

After obtaining written informed consent, the peripheral blood sample of the proband was collected for whole exome sequencing (WES) to seek for potential genetic defects. A small heterozygous 9p21.3 deletion involving *CDKN2A*, *CDKN2A-DT*, and exon 1 of *ANRIL* was suspected from the decreased read number in the WES data (**Figure 2A**). The deletion was predicted to be 28 kb based on the spanning of captured exons (human assembly GRCh37/hg19 chr9:21,967,137_21,995,160). For confirmation, five pairs of primers against the *CDKN2A*-coding region were designed for quantitative PCR (qPCR) analysis, showing that all the amplified fragments were decreased to half levels of the internal control (**Figure 2B**). According to the American College of Medical Genetics and Genomics (ACMG) guideline, the deletion was ranked as “pathogenic” for completely covering the established haploinsufficiency gene *CDKN2A*, which is related to the susceptibility to melanoma and neural system tumor syndrome (OMIM 155755), melanoma-pancreatic cancer syndrome (OMIM 606719), and melanoma, cutaneous malignant, 2 (OMIM 155601). No variation was found in the *neurofibromatosis type 1* and *2* (*NF1* and *NF2*) genes in the WES data, and MLPA (SALSA MLPA Probemix P081, P082, and P044) was also performed to exclude exon deletions or duplications in *NF1* and *NF2* (data not shown). We have also carefully inspected other putative variants of known genes that are correlated with neurofibroma and melanoma in the raw data of WES. All the variants with allele frequency below 0.01 are listed in **Supplementary Table S1**. None of them is ranked as “pathogenic” or “likely pathogenic”. Collectively, the heterozygous 9p21.3 microdeletion involving *CDKN2A*, *CDKN2A-DT*, and exon 1 of *ANRIL* should be responsible for the multiple PNFs in the proband. The patient was suggested to avoid smoking and alcohol, take sun protection, receive human papillomavirus (HPV) vaccination, and monitor tumors with regular follow-up.

The couple requested for prenatal diagnosis for this torturous disease, and amniocentesis was performed at her 18 gestational weeks. Karyotype analysis of amniocytes displayed normal results, whereas copy number variation-sequencing (CNV-seq) showed a 0.1-Mb heterozygous deletion on chromosome 9p21.3 (GRCh37/hg19 chr9:21,900,000_22,000,000) (**Figure 2C**), covering the last exon of *MTAP*, *CDKN2A*, and *CDKN2A-DT* and exon 1 of *ANRIL*. The WES data of *MTAP* were rechecked in the gravida, showing normal read numbers of exon 8 and the 3'UTR region, which was confirmed by qPCR (data not shown). Considering the different resolution of WES and CNV-seq technologies, the deletion in the fetus should be maternally inherited. For posttest genetic counseling, the patient was informed of an affected fetus with the early-onset cancer predisposition syndrome. After in-depth consideration about the increased risk of premalignant and malignant tumors, the couple terminated the pregnancy at 20 gestational weeks with informed choice. For 50% chance of inheriting the small deletion, preimplantation genetic testing (PGT) was suggested for her next pregnancy using the remaining DNA of amniocytes for haplotype construction. By the time of a recent follow-up, the woman had been pregnant after PGT. Amniocentesis was also performed for prenatal diagnosis, showing normal results of karyotyping and CNV-seq.

A total of eight cases with 9p21.3 microdeletions have been reported in literature (including the present case) (**Figure 2D**). They are from different ethnic origins, among which Caucasian was the most frequent (3/8). The deletion sizes ranged from 28 kb to more than 2 Mb. All the deletions encompassed the entire *CDKN2A*/*CDKN2B*/*ANRIL* locus and the flanking genes except for our case. Phenotypically, melanoma was characteristic that occurred in 75% (6/8) of the cases. The two cases without melanoma were from Singapore and China. Other cutaneous implications included atypical/dysplastic nevi or junctional naevus (3/8), and squamous papilloma or carcinoma (3/8). Neural system tumors of various types occurred in all the cases, including neurofibromas (4/8), astrocytoma (4/8), peripheral nerve sheath tumor (2/8), and meningiomas (1/8). Moreover, other tumors were also reported in one patient including extraskeletal osteosarcoma, low-grade myofibroblastic sarcoma, and lymphoepithelial carcinoma. Comparatively, our case with the smallest deletion had the mildest symptom of PNF only and, together with the other Eastern Asian origin, had no melanoma.

## Discussion

Germline 9p21.3 deletions involving the contiguous *CDKN2A*/*CDKN2B*/*ANRIL* region exhibit cancer predisposition syndrome with characteristics overlapping NF1, familial atypical multiple mole melanoma syndrome (FAMMM), and Li–Fraumeni syndrome (LFS) (7, 8). The proband was previously thought to have NF1 for her early onset and rapid increase of neurofibromas. Yet, no cafe-au-lait spots, axillary/inguinal freckling, or Lisch nodule were observed after examination. No small sequence variation or exon deletion/duplication was found in *NF1* and *NF2* by WES and MLPA. By going through the WES data prudently, we found decreased reads of the contiguous *CDKN2A*, *CDKN2A-DT*, and exon 1 of *ANRIL*, which support a small heterozygous 9p21.3 deletion spanning 28 kb. The core protein-coding *CDKN2A* gene was validated for heterozygous deletion by qPCR using five pairs of primers, which substantiated the microdeletion as “pathogenic” for covering this established haploinsufficiency gene. It is usually challenging to recognize small CNVs by WES through short-read next generation. For patients with multiple neurofibromas, there is a necessity to look over the captured CNV on 9p21.3.

Our case is the smallest germline 9p21.3 deletion involving *CDKN2A* but not *CDKN2B*. It is well known that *CDKN2A* is the most frequently mutated high-risk gene in familial melanoma. Carriers have a risk of melanoma that is greater than a 65-fold increase compared with the normal population, and a lifetime penetrance for melanoma of 60% to 90% (11). Owning to the common embryologic precursor of the neuroectoderm, some germline *CDKN2A* mutations are prone to develop both melanomas and neural system tumors (12, 13). It is interesting that our patient was found with neither melanoma nor any presymptom of dysplastic nevus. No other cutaneous tumors were observed, either. By reviewing literature, only one Singaporean case with germline 9p21.3 deletion involving *CDKN2A*, *CDKN2B*, and partial *MTAP* and *ANRIL* genes had no melanoma but squamous cell carcinoma in the head and neck (8). The additive role of *CDKN2B* and other contiguous genes might contribute to the development of melanoma, since p15 expression encoded by *CDKN2B* has been shown to differentiate nevus from melanoma (14). Meanwhile, the ethnic background of Eastern Asian origin, environmental factors, and the strong willingness to take sun protection could not be ignored. More Asian cases are needed for further correlation, and the possibility of late-onset melanoma in these cases cannot be ruled out.

By literature reviewing, all the previously reported cases with germline 9p21.3 deletion encompassed the entire *CDKN2A*/*CDKN2B*/*ANRIL* locus. The tumor spectrum was broad, including but not limited to malignant melanomas and neural system tumors (astrocytoma, neurofibromas, schwannomas, and meningiomas). An Iraqi case with 1.7-Mb deletion involving *FOCAD*, *IFN* gene cluster, *MTAP*, *CDKN2A*, *CDNK2A-DT*, *CDKN2B*, and *ANRIL* had multiple malignancies including melanoma, extraskeletal osteosarcoma, lymphoepithelial carcinoma, low-grade myofibroblastic sarcoma, anaplastic pleomorphic xanthoastrocytoma, astrocytoma, and 16 benign or premalignant tumors (10), while the Italian case with a larger 2.135-Mb deletion presented with fewer tumors including melanomas, dysplastic nevi, and anaplastic astrocytoma (6). The tumor proneness could not be simply related to the size of the deletions, especially for genes with limited knowledge of the associated cancer risks. The interaction with other genomic loci may also play a role. Comparatively, our patient carried the smallest 28-kb deletion involving *CDKN2A*, *CDNK2A-DT*, and the first exon of *ANRIL*. *CDNK2A-DT* is a small antisense non-coding RNA gene located at the 3'UTR region of *CDKN2A*, with an unknown role in tumorigenesis. *ANRIL* is a large antisense non-coding RNA gene (126-kb region, consisting of 19–21 exons) with the first exon located in the promoter region of *CDKN2A*, regulating cancer progression through epigenetic silencing of other genes in this cluster (15). It is not sure whether the deletion of its first exon could completely disrupt the transcription of *ANRIL* and abolish its role in tumorigenesis. Our proband had only PNF with benign nature at present, strengthening the contributive effect of at least *CDKN2B* and possibly *ANRIL* in tumorigenesis.

It is a clinical challenge for posttest genetic counseling of cancer predisposition syndromes that related to reproductive choice. The ACMG guideline for prenatal WES recommended reporting of some late-onset genes including cancer genes as secondary findings. PNF is a kind of histologically benign peripheral nerve sheath tumor. However, it causes functional damage, deformities, and pain and increases the risk of transformation into atypical neurofibromas (ANF) and further into aggressive malignant peripheral nerve sheath tumor (MPNST) (16, 17). Our patient had suffered a lot including 11 times of surgical resections, the requirement for prenatal diagnosis by the couple was allowed, and the informed choice of termination of the pregnancy of an affected fetus was also respected. Since the gravida was adopted with unavailability of pedigree tracing and deletion was much smaller than 1 Mb, haplotype construction was achieved using the remaining amniocyte DNA and a pregnancy of an unaffected fetus was obtained after PGT and subsequent prenatal diagnosis.

In conclusion, we described the tumorous characteristics of multiple PNFs without melanoma in a pregnant woman carrying the smallest germline 9p21.3 microdeletion involving *CDNK2A* but not *CDNK2B*. Our data expand the genotype–phenotype correlation of 9p21.3 microdeletion, highlight 9p21.3 deletion as a potential differential diagnosis for neurofibroma, and emphasize the importance of CNV analysis on the WES data wherein small deletions might be easily overlooked. We also share our experience in prenatal diagnosis, genetic counseling, and reproductive choice for the affected individuals.

## Data Availability

The datasets presented in this article are not readily available due to privacy-related concerns. Requests to access the datasets should be directed to the corresponding authors.
