# Supplementary material for: Case report: The smallest 9p21.3 microdeletion involving CDKN2A but not CDKN2B causes multiple plexiform neurofibromas
Source: Front Oncol. 2025 Feb 19;15:1437093. doi: 10.3389/fonc.2025.1437093 (PMC11880698; doi:10.3389/fonc.2025.1437093)
Supplement: Supplementary file 1 [file Table1.docx]

Supplementary Table 1. Germline variants of genes related with neurofibroma and melanoma in the proband.

| Gene | Variant | Allele frequency | In silico prediction | Rank of pathogenicity |
| --- | --- | --- | --- | --- |
| *HRAS* | NM_005343.4 c.*1558C>G | 0.000624805 | Unknown | Uncertain |
| *NF1* | NM_000267.3 c.7675+147_7675+148del | - | Unknown | Uncertain |
| *NF1* | NM_000267.3 c.*584G>A | - | Unknown | Uncertain |
| *BRCA1* | NM_007294.4 c.-1395C>T | 0.00513479 | Unknown | Uncertain |
| *BAP1* | NM_004656.4 c.534C>T (p.Gly178=) | 0.0065 | Unknown | Likely benign |
| *BAP1* | NM_004656.4 c.912C>A (p.Ala304=) | 0.0058 | Unknown | Likely benign |
